# Prognostic value of a novel indicator integrating C-reactive protein-triglyceride glucose index and coronary physiological burden in patients undergoing PCI: a prospective cohort study

**DOI:** 10.3389/fnut.2026.1874235

**Published:** 2026-07-31

**Authors:** Xinjun Lin, Yuanming Yan, Hui Chen, Jiaxin Zhong, Yuxiang Chen, Beilei Li, Lin Fan, Yukun Luo, Lianglong Chen, Qin Chen

**Affiliations:** 1 Department of Cardiology, Fujian Medical University Union Hospital, Fuzhou, Fujian, China; 2 Fujian Cardiovascular Medicine Center, Fuzhou, Fujian, China; 3 Fujian Institute of Coronary Artery Disease, Fuzhou, Fujian, China; 4 Fujian Cardiovascular Research Center, Fuzhou, Fujian, China; 5 Fujian Medical University Heart Center, Fuzhou, Fujian, China; 6 Department of Emergency, Fujian Medical University Union Hospital, Fuzhou, Fujian, China; 7 Department of Geriatric Medicine, Fujian Medical University Union Hospital, Fuzhou, Fujian, China

**Keywords:** C-reactive protein-triglyceride glucose index, inflammation, insulin resistance, major adverse cardiovascular and cerebrovascular events, percutaneous coronary intervention, quantitative flow ratio

## Abstract

**Background:**

Patients with coronary artery disease (CAD) continue to face residual cardiovascular risk despite receiving guideline-directed optimal therapy. Both systemic metabolic-inflammatory dysregulation, reflected by the C-reactive protein-triglyceride glucose index (CTI), and coronary physiological burden, assessed by the quantitative flow ratio (QFR), are critical determinants of prognosis. However, the combined prognostic value of these two factors in patients after percutaneous coronary intervention (PCI) remains unclear. This study aimed to evaluate the prognostic value of an integrated index combining CTI with residual coronary physiological burden in patients undergoing PCI.

**Methods:**

This single-center registry study (ChiCTR2100042363) consecutively enrolled 1,468 patients with CAD who underwent PCI between March 2021 and February 2022. A novel integrated index, μCTI, was derived from the CTI and the sum of the residual QFR in the three major coronary vessels. The primary endpoint was major adverse cardiovascular and cerebrovascular events (MACCE), defined as a composite of all-cause death, non-fatal myocardial infarction (MI), ischemia-driven revascularization (IDR), or stroke.

**Results:**

During a median follow-up of 24 months, a total of 185 MACCE (12.6%) occurred. In multivariable Cox regression, each standard deviation increase in μCTI was independently associated with a 79% rise in MACCE risk [adjusted hazard ratio (HR) = 1.79, 95% CI: 1.54–2.07, *p* < 0.001]. Furthermore, MACCE risk progressively increased across ascending μCTI quartiles (P for trend < 0.001). Restricted cubic spline (RCS) analysis revealed a linear relationship between μCTI and MACCE risk (P for nonlinear = 0.058).

**Conclusion:**

The composite μCTI acts as an independent predictive biomarker and delivers supplementary risk stratification information for patients after PCI. This combined index could serve as an auxiliary tool to identify individuals at elevated risk of MACCE beyond single metabolic-inflammatory or coronary physiological indicators.

## Graphical abstract

**Figure:**
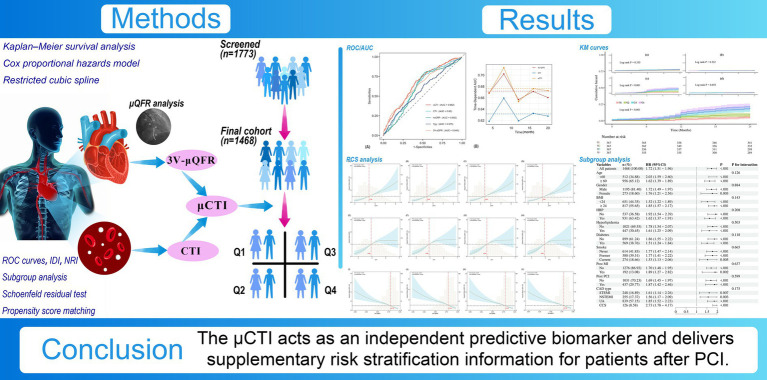

## Introduction

Coronary artery disease (CAD) remains a leading global cause of mortality and disability (1–3). Even after patients receive guideline-directed medical therapy and percutaneous coronary intervention (PCI), persistent residual cardiovascular events are commonly observed (4).

Current evidence suggests that the synergistic interaction between inflammation and insulin resistance (IR) constitutes a significant pathological underpinning of residual cardiovascular risk (5). The triglyceride-glucose (TyG) index is a simple surrogate marker of IR and has been associated with coronary lesion severity and long-term cardiovascular risk (6–8). A recent cohort study focusing on chronic total occlusion PCI patients further reported that elevated TyG levels independently predicted contrast-induced nephropathy, which supports the clinical value of this metabolic marker in complex high-risk revascularization procedures (9). C-reactive protein (CRP), the most widely used clinical indicator of systemic inflammation, also independently predicts the occurrence and adverse prognosis of CAD (10–13). Metabolic and inflammatory signaling pathways interact to form a self-reinforcing pathogenic cycle that sustains vascular injury (14). To simultaneously reflect insulin resistance and systemic inflammation, the C-reactive protein-triglyceride glucose index (CTI) was developed (15). Data from large nationwide prospective cohorts demonstrate that CTI performs better than isolated TyG or hs-CRP for predicting cardiovascular events and all-cause mortality (16–18).

The clinical outcome of CAD is determined not only by systemic pathophysiological conditions but also, and more directly, by coronary physiological burden (19). Quantitative flow ratio (QFR) can be calculated from standard coronary angiograms without additional pressure guidewires (20–22). Murray’s law-based μQFR can accurately identify hemodynamically significant stenosis and guide revascularization decisions, as recommended by international cardiovascular guidelines (23, 24). Meanwhile, the residual three-vessel QFR (3 V-QFR) has been proven to be related to the clinical outcome (25, 26).

However, current clinical risk assessment often regards systemic biomarkers and coronary physiological evaluation as separate domains. Moreover, most studies on CTI have been conducted in general population cohorts, whereas evidence in high-risk post-PCI patients remains limited (18, 27–29). Moreover, research integrating CTI and μQFR for prognostic evaluation in patients with established CAD is still limited. Therefore, this study aimed to evaluate the prognostic value of an integrated index combining CTI with residual coronary physiological burden in patients undergoing PCI.

## Methods

### Study population

The data for this study were sourced from a single-center, observational registry study (Chinese Clinical Trial Registry number: ChiCTR2100042363), which consecutively enrolled adult patients with CAD who underwent PCI at our center from March 2021 to February 2022. Clinical exclusion criteria were: (1) severe renal insufficiency (serum creatinine >150 mmol/L or estimated glomerular filtration rate <45 mL/min/1.73 m^2^); (2) concurrent malignancy; (3) history of coronary artery bypass grafting (CABG); (4) life expectancy <2 years; (5) pregnancy. Angiographic exclusion criteria included: (1) lesion located on the vessel with a myocardial bridge; (2) poor angiographic quality or significant vessel overlap/tortuosity prevented accurate QFR analysis. The study was conducted in accordance with the principles of the Declaration of Helsinki and was approved by the Ethics Committee of Fujian Medical University Union Hospital (2021KY017). Written informed consent was obtained from all participants.

### μQFR analysis procedure

All μQFR measurements were conducted according to a standardized protocol with systematic quality control throughout the analysis process. μQFR analysis was performed by two interventional cardiologists who were blinded to patient clinical information, qualified in interventional therapy, and certified in computational coronary physiology training. They utilized validated QFR system software (AngioPlus Core, Pulse Medical Imaging Technology, Shanghai, China). The analysis adhered to the following steps: A single angiographic projection image that best visualized the coronary stenosis was selected and imported into the QFR system. The software automatically delineated the lumen contours of the target vessel and its branches with a diameter ≥1 mm. Subsequently, one frame exhibiting optimal contrast filling and complete vessel lumen contours was chosen as the key frame for analysis. The software automatically traced the borders of the target vessel and its major branches. If the quality of the automatic tracing was suboptimal, manual correction was performed. μQFR computation employed a contrast-flow velocity-based model and a pressure-drop algorithm derived from fluid dynamics equations. The μQFR values for the target vessel and its branches were then calculated separately. μQFR measurements for each of the three main coronary vessels were recorded.

### Definitions

Coronary functional burden was quantified by the sum of residual 3 V-μQFR after PCI. A coronary functional adjustment factor (CFAF), scaled from 0 to 1, was calculated as: CFAF = (3 V-μQFR) / 3. A CFAF value closer to 1 indicates a lower overall coronary physiological burden. The CTI was calculated as follows: CTI = 0.412 × ln(CRP [mg/L]) + ln(TG [mg/dL] × FBG [mg/dL]) / 2 (15–17, 24, 25). To integrate systemic metabolic-inflammatory status with coronary functional burden, a novel combined index, μCTI (combined 3 V-μQFR and CTI), was constructed using the formula: μCTI = CTI / CFAF. A given level of systemic metabolic-inflammatory activity may have different prognostic implications depending on the residual physiological status of the coronary circulation. This index aims to quantify the level of systemic metabolic-inflammatory load per unit of coronary physiological functional reserve. Statistically, dividing CTI by CFAF allowed CTI to be weighted by residual coronary physiological impairment, so that higher μCTI values indicate greater metabolic-inflammatory burden in the context of more pronounced residual coronary functional abnormality.

### Data collection and clinical outcomes

Patient clinical characteristics, laboratory tests, interventional procedural data, and angiographic images were collected from the electronic medical record system. Patients with missing baseline data required for the calculation of μCTI were excluded from the study. For other baseline covariates with a small proportion of missing values, multiple imputation based on random forest was used to impute missing data before statistical analysis. Study angiographic images were anonymized and analyzed by a dedicated analyst. Clinical follow-up was conducted at 30 days post-procedure, and subsequently at 6, 12, and 24 months. Follow-up data were collected via outpatient visit records, hospitalization information, or telephone interviews. Follow-up concluded on February 25, 2024, or at the time of an endpoint event. Endpoint events were adjudicated by a clinical endpoint committee blinded to group allocation. The primary endpoint of this study was the occurrence of post-PCI MACCE during follow-up, defined as a composite of all-cause death, non-fatal myocardial infarction (MI), ischemia-driven revascularization (IDR), or stroke. Clinical outcomes were defined according to the Academic Research Consortium standards (30). MI was defined based on the Fourth Universal Definition (31). The time-to-event was defined as the interval from the date of the PCI to the date of a subsequent event.

### Statistical analysis

Continuous variables are presented as mean ± standard deviation or median (IQR), depending on their distribution. Comparisons across μCTI quartiles were performed using one-way analysis of variance or the Kruskal–Wallis test, as appropriate, whereas Student’s t-test or the Mann–Whitney U test was used for two-group comparisons. Categorical variables are presented as frequency (%), with between-group comparisons performed using the chi-square test or Fisher’s exact test. To evaluate the predictive ability of each indicator for MACCE, receiver operating characteristic (ROC) curve analysis was used to determine the optimal cutoff value for MACCE prediction in different models, and the area under the curve (AUC) was calculated. DeLong’s test was applied to compare differences in AUC between models, and the net reclassification improvement (NRI) and integrated discrimination improvement (IDI) were used to assess improvement in model predictive performance. Comparison of endpoint event rates between groups was performed using the chi-square test. When overall significance was detected for an outcome, post-hoc pairwise comparisons of risk categories were performed with Bonferroni correction. The significance threshold was adjusted by dividing the standard level (0.050) by the number of pairwise comparisons performed. Identical symbols within the same row indicate statistically significant differences between the corresponding groups after Bonferroni-corrected pairwise post-hoc comparisons. Trend analysis was performed using the Cochran-Armitage test. Survival curves were plotted using the Kaplan–Meier method, with between-group differences assessed by the log-rank test and adjusted for multiple comparisons using the Hommel method. Multiple multivariable Cox proportional hazards (PH) regression models were constructed to evaluate the independent association of CTI and μCTI with MACCE risk, and hazard ratio (HR) with 95% confidence intervals (CI) were calculated. The variance inflation factor (VIF) was used to test for multicollinearity. Restricted cubic spline (RCS) analysis was employed to examine the dose–response relationship between μCTI, CTI, 3 V-μQFR, and MACCE risk. Consistency analysis was performed within predefined clinical subgroups, and its significance was tested using interaction terms.

Finally, to verify the robustness of the results, the following sensitivity analyses were performed: (1) PH assumption testing using Schoenfeld residuals and residual plots, with the PH assumption accepted if *p* > 0.05; (2) Multigroup propensity score matching to balance baseline characteristics; (3) Exclusion of patients with left ventricular ejection fraction < 40%; (4) Exclusion of patients with baseline total occlusion lesions (including chronic total occlusion and acute occlusion); (5) Restriction of the analysis to patients who achieved functional complete revascularization in all three vessels; (6) Worst-case scenario imputation analysis for patients lost to follow-up. Data analysis was conducted using R version 4.3.2 and Python version 3.10.6. All tests were two-sided, and *p* < 0.05 was considered statistically significant.

## Results

### Baseline characteristics

**Figure 1:**
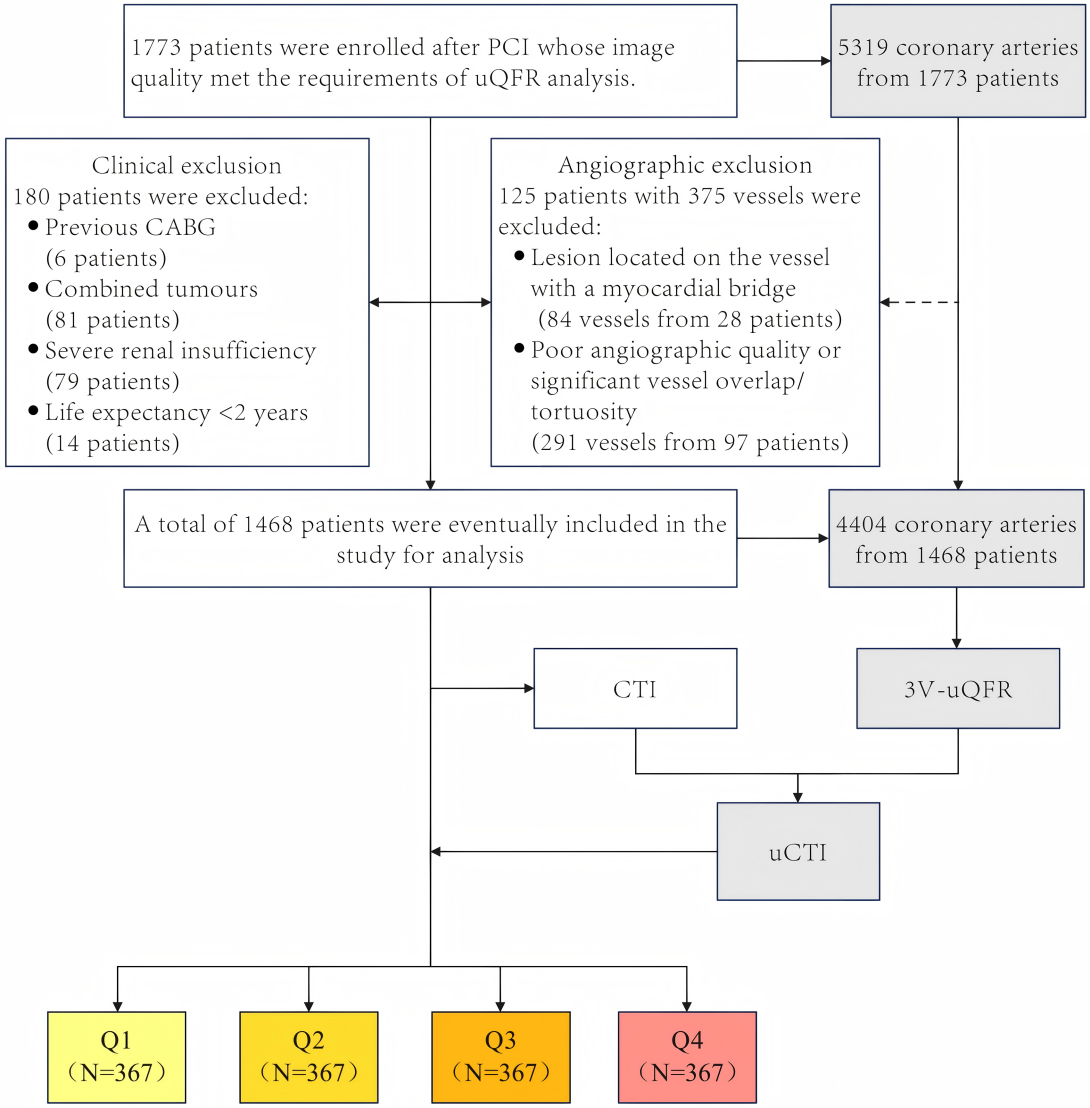
Study flowchart. PCI, percutaneous coronary intervention; μQFR, Murray’s law-based quantitative flow ratio; CABG, coronary artery bypass grafting; CTI, C-reactive protein-triglyceride glucose index; 3 V-μQFR, three-vessel μQFR; μCTI, combined 3 V-μQFR and CTI index; Q, quartile.

This study ultimately included 1,468 patients (Figure 1), with a median follow-up time of 24 months. During follow-up, 48 patients were lost. Patients were divided into four groups based on μCTI quartiles (Q1–Q4, quartile cut-offs: 4.797, 5.395, 6.071; *n* = 367 per group). Their baseline characteristics are presented in Table 1. In brief, the mean age was 63.57 ± 10.79 years, and 18.60% were female. Indicators related to μCTI (μCTI, CTI, and 3 V-μQFR) all showed statistically significant differences across the groups (all *p* < 0.001). Among the clinical characteristics, BMI, diabetes, previous PCI, and clinical diagnosis also showed significant differences between the groups (all *p* < 0.001). Furthermore, most laboratory parameters, except for serum creatinine, exhibited statistically significant intergroup differences (all *p* < 0.001). Medication use of statins, beta-blockers, renin-angiotensin system inhibitors (RASi), and sodium-glucose cotransporter 2 inhibitors (SGLT2i) also showed differences (*p* < 0.05). No significant differences were observed in other demographic features, medication use, or interventional data. Detailed baseline characteristics for all 4,404 vessels are provided in Supplementary Table S1. Additionally, with the exception of clinical diagnosis, no significant differences were found in the baseline characteristics between patients who completed follow-up (n = 1,420) and those lost to follow-up (*n* = 48) (Supplementary Table S2), indicating that loss to follow-up did not introduce substantial bias.

**Table 1:** Baseline characteristics of participants grouped by quartiles of μCTI.

Characteristic	Overall (*N* = 1,468)	Q1 (*N* = 367)	Q2 (*N* = 367)	Q3 (*N* = 367)	Q4 (*N* = 367)	*p* value
Clinical features						
Age, years	63.57 ± 10.79	64.19 ± 9.72	63.69 ± 10.44	63.67 ± 10.73	62.74 ± 12.11	0.326
Female, *n* (%)	273 (18.60)	68 (18.53)	73 (19.89)	71 (19.35)	61 (16.62)	0.685
BMI, kg/m^2^	24.49 ± 3.16	23.87 ± 3.09	24.36 ± 3.09	24.92 ± 3.29	24.83 ± 3.06	<0.001
Diabetes, *n* (%)	569 (38.76)	96 (26.16)	130 (35.42)	160 (43.60)	183 (49.86)	<0.001
Hypertension, *n* (%)	931 (63.42)	215 (58.58)	230 (62.67)	248 (67.57)	238 (64.85)	0.077
Previous MI, *n* (%)	192 (13.08)	46 (12.53)	49 (13.35)	57 (15.53)	40 (10.90)	0.309
Previous PCI, *n* (%)	437 (29.77)	108 (29.43)	117 (31.88)	123 (33.51)	89 (24.25)	0.035
Smoking, *n* (%)						0.159
Never	614 (41.83)	163 (44.41)	145 (39.51)	164 (44.69)	142 (38.69)	
Former	274 (18.66)	77 (20.98)	70 (19.07)	64 (17.44)	63 (17.17)	
Current	580 (39.51)	127 (34.60)	152 (41.42)	139 (37.87)	162 (44.14)	
Clinical diagnosis						<0.001
CCS, *n* (%)	126 (8.58)	37 (10.08)	43 (11.72)	29 (7.90)	17 (4.63)	
UA, *n* (%)	839 (57.15)	248 (67.57)	225 (61.31)	219 (59.67)	147 (40.05)	
NSTEMI, *n* (%)	255 (17.37)	51 (13.90)	55 (14.99)	60 (16.35)	89 (24.25)	
STEMI, *n* (%)	248 (16.89)	31 (8.45)	44 (11.99)	59 (16.08)	114 (31.06)	
Laboratory and examination data						
LDL-C (mmol/L)	2.85 ± 1.24	2.68 ± 1.43	2.78 ± 1.20	2.87 ± 1.16	3.06 ± 1.13	<0.001
TG (mmol/L)	1.71 ± 1.24	1.16 ± 0.47	1.55 ± 0.73	1.93 ± 1.52	2.17 ± 1.57	<0.001
TC (mmol/L)	4.37 ± 1.29	4.08 ± 1.12	4.30 ± 1.31	4.46 ± 1.34	4.66 ± 1.29	<0.001
HDL-C (mmol/L)	1.07 ± 0.31	1.15 ± 0.31	1.10 ± 0.30	1.04 ± 0.29	1.00 ± 0.30	<0.001
Scr (umol/L)	77.85 ± 17.77	76.92 ± 16.84	76.68 ± 16.53	77.84 ± 18.33	79.96 ± 19.16	0.051
FBG	6.18 ± 2.16	5.24 ± 0.98	6.05 ± 1.94	6.16 ± 1.98	7.25 ± 2.82	<0.001
HbAlc (%)	6.71 ± 1.44	6.22 ± 0.94	6.61 ± 1.29	6.78 ± 1.39	7.24 ± 1.80	<0.001
LVEF (%)	60.69 ± 11.07	61.91 ± 10.77	61.59 ± 10.73	61.59 ± 10.73	58.42 ± 11.45	<0.001
hs-CRP	1.43 (0.52, 5.50)	0.41 (0.30, 0.57)	0.90 (0.52, 1.55)	2.99 (1.52, 5.71)	10.30 (4.82, 20.55)	<0.001
CTI	5.02 ± 0.72	4.19 ± 0.24	4.72 ± 0.24	5.25 ± 0.30	5.91 ± 0.50	<0.001
μCTI	5.49 ± 0.91	4.45 ± 0.24	5.09 ± 0.17	5.71 ± 0.20	6.70 ± 0.64	<0.001
Medication						
Statins, *n* (%)	1,453 (98.98)	361 (98.37)	361 (98.37)	364 (99.18)	367 (100.00)	0.047
Beta-blocker, *n* (%)	1,155 (78.68)	266 (72.48)	292 (79.56)	294 (80.11)	303 (82.56)	0.006
RASi, *n* (%)	940 (64.03)	209 (56.95)	224 (61.04)	244 (66.49)	263 (71.66)	<0.001
CCB, *n* (%)	487 (33.17)	113 (30.79)	129 (35.15)	123 (33.51)	122 (33.24)	0.658
SGLT2i	246 (16.76)	35 (9.54)	53 (14.44)	67 (18.26)	91 (24.80)	<0.001
Interventional data						
Angiographic disease severity						0.238
1-vessel disease	1,083 (73.77)	281 (76.57)	272 (74.11)	270 (73.57)	260 (70.84)	
2-vessel disease	315 (21.46)	75 (20.44)	73 (19.89)	83 (22.62)	84 (22.89)	
3-vessel disease	70 (4.77)	11 (3.00)	22 (5.99)	14 (3.81)	23 (6.27)	
3 V-μQFR	2.76 ± 0.17	2.83 ± 0.09	2.78 ± 0.11	2.76 ± 0.14	2.66 ± 0.24	<0.001

Data are presented as mean ± SD, median (IQR), or *n* (%). All patients received standardized dual antiplatelet therapy after PCI. μCTI, combined 3 V-μQFR and CTI index; Q, quartile; BMI, body mass index; MI, myocardial infarction; PCI, percutaneous coronary intervention; CCS, chronic coronary syndromes; UA, unstable angina; NSTEMI, non-ST-segment elevation myocardial infarction; STEMI, ST-segment elevation myocardial infarction; LDL-C, low-density lipoprotein cholesterol; TG, triglyceride; TC, total cholesterol; HDL-C, high-density lipoprotein cholesterol; Scr, serum creatinine; FBG, fasting blood glucose; HbA1c, glycated hemoglobin; LVEF, left ventricular ejection fraction; hs-CRP, high-sensitivity C-reactive protein; CTI, C-reactive protein-triglyceride glucose index; RASi, renin-angiotensin system inhibitors; CCB, calcium channel blocker; SGLT2i, sodium-glucose cotransporter 2 inhibitor; 3 V-μQFR, three-vessel μQFR; μQFR, quantitative flow ratio based on Murray’s law.

### Discriminative and incremental value of μCTI in predicting MACCE

**Figure 2:**
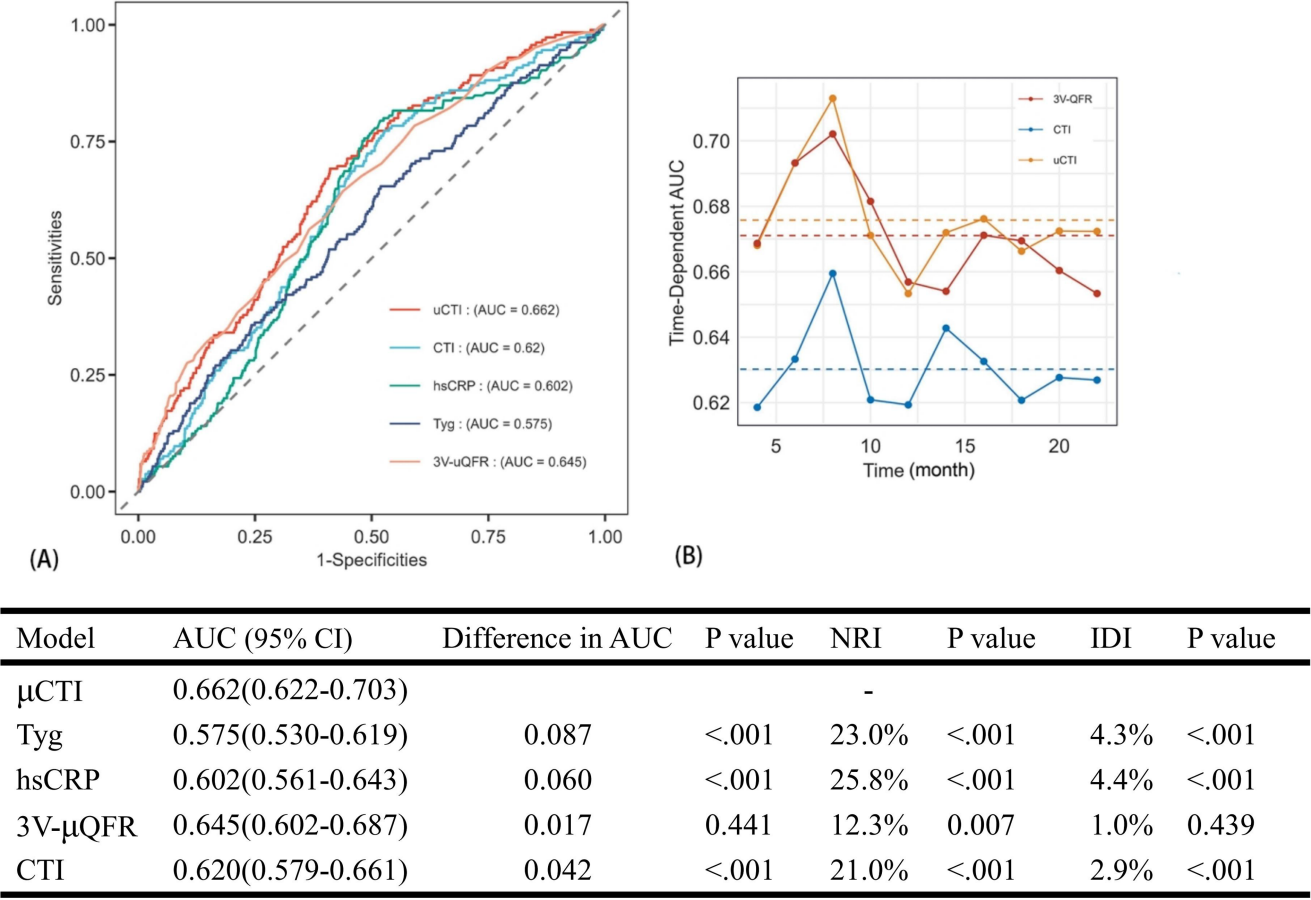
ROC curves and time-dependent AUC analysis for MACCE prediction by different models. **(A)** ROC curves for predicting MACCE. **(B)** Time-dependent AUC analysis over the follow-up period. The table summarizes the AUC, difference in AUC, NRI, and IDI for each model compared to the *μ*CTI model. MACCE, major adverse cardiovascular and cerebrovascular events; AUC, area under the curve; *μ*CTI, combined 3 V-*μ*QFR and CTI index; TyG, triglyceride-glucose index; hs-CRP, high-sensitivity C-reactive protein; 3 V-*μ*QFR, three-vessel *μ*QFR; CTI, C-reactive protein-triglyceride glucose index; NRI, net reclassification improvement; IDI, integrated discrimination improvement.

The predictive performance of μCTI for MACCE was assessed using ROC curves (Figure 2A). The AUC for the μCTI model in predicting MACCE was 0.662 (95% CI: 0.622–0.703). Comparison via DeLong’s test revealed that the AUC of μCTI was significantly higher than that of the CTI index (difference in AUC: 0.042, *p* < 0.001), the TyG index (difference in AUC: 0.087, *p* < 0.001), and hs-CRP (difference in AUC: 0.060, *p* < 0.001). Further NRI and IDI analyses demonstrated that incorporating μCTI into the prediction model yielded significant improvements in predictive capability compared to models using the TyG index, hs-CRP, or CTI alone (all *p* < 0.001) (Figure 2). DeLong’s test and IDI analysis indicated no significant difference in discriminative ability between the μCTI model and the 3 V-μQFR model. However, μCTI demonstrated significant incremental value in risk reclassification (NRI improvement of 12.3%, *p* = 0.007), suggesting that it may provide additional information for stratifying patients into different risk categories. Time-dependent ROC curve analysis showed that μCTI maintained relatively stable predictive performance across the follow-up period, with their overall AUCs being higher than those of the other models (Figure 2B). These results indicate that the μCTI model provided additional prognostic information, particularly in terms of risk reclassification.

### Association between μCTI and clinical outcomes

**Figure 3:**
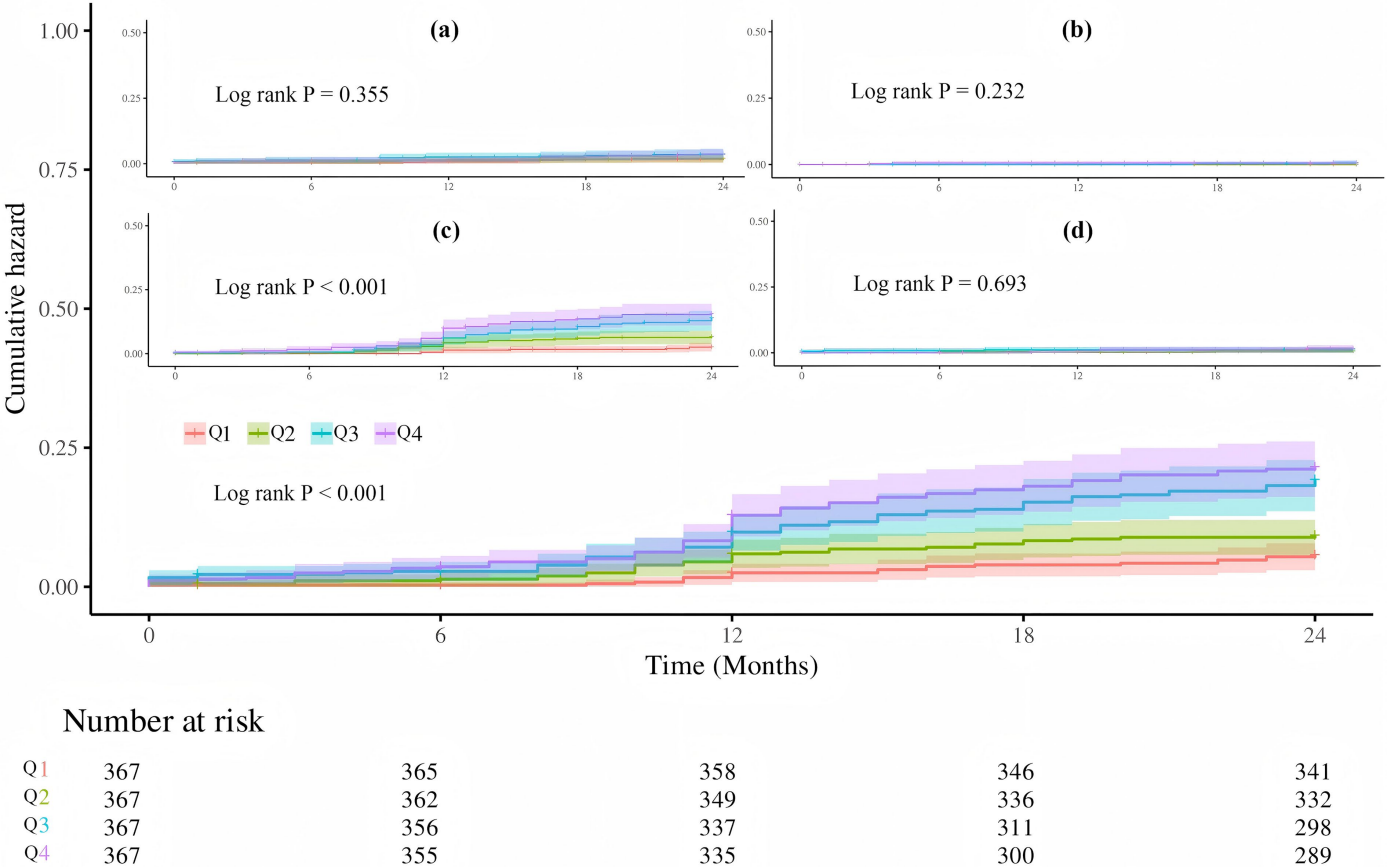
Kaplan–Meier curves for cumulative incidence of MACCE and its components according to *μCTI* quartiles. Kaplan–Meier curves are shown for **(a)** All-cause death, **(b)** MI, **(c)** IDR, **(d)** Stroke, and the composite endpoint of MACCE across quartiles (Q1–Q4) of the *μCTI* index. MACCE, Major adverse cardiovascular and cerebrovascular events; *μCTI*, Combined 3 V-*μQFR* and CTI index; Q, Quartile; MI, Myocardial infarction; IDR, Ischemia-driven revascularization.

Kaplan–Meier survival curves visually depict the cumulative incidence of clinical events across μCTI quartile groups (Figure 3). For the primary composite endpoint MACCE, the survival curves showed clear separation, indicating a significant decline in event-free survival with increasing μCTI quartiles (Log-rank *p* < 0.001). Pairwise comparisons of the MACCE survival curves were performed with adjustment for multiple testing using the Hommel method (Table 2). The results showed statistically significant differences between the Q1 group and the Q3/Q4 groups (adjusted *p* < 0.001), and similarly between the Q2 group and the Q3/Q4 groups (adjusted *p* = 0.002). However, the differences between Q1 and Q2, and between Q3 and Q4, did not reach statistical significance.

**Table 2:** Pairwise comparisons of MACCE risk across μCTI quartiles using Hommel’s adjustment method.

Row name	Q1	Q2	Q3
Q2	0.160	–	–
Q3	<0.001	0.002	–
Q4	<0.001	0.002	0.490

*p* values are adjusted for multiple comparisons using the Hommel method. Q1–Q4 represent quartiles of μCTI. MACCE, major adverse cardiovascular and cerebrovascular events; μCTI, combined 3 V-μQFR and CTI index.

Separate analysis of individual MACCE components (Table 3) revealed that the incidence rates of MACCE and revascularization events differed significantly across μCTI groups (*p* < 0.001) and exhibited a significant gradient (P for trend < 0.001). The pattern of differences in pairwise comparisons with Bonferroni correction was similar to that obtained with the Hommel method described above. In contrast, the incidence rates of all-cause death, MI, and stroke were low across all groups, and no significant intergroup differences were observed. These analyses indicate that the intergroup differences in MACCE incidence may be primarily driven by IDR. The association between μCTI and MACCE risk can therefore be largely attributed to its strong link with IDR.

**Table 3:** Association between μCTI quartiles and incidence of MACCE and its components.

Endpoint events	Total (*N* = 1,468)	Q1 (*N* = 367)	Q2 (*N* = 367)	Q3 (*N* = 367)	Q4 (*N* = 367)	*p* value	*p* value for trend
MACCE	185 (12.6)	20 (5.4)^a,b^	32 (8.7)^c,d^	63 (17.2)^a,c^	70 (19.1)^b,d^	<0.001	<0.001
All-cause death	38 (2.6)	7 (1.9)	7 (1.9)	12 (3.3)	12 (3.3)	0.440	0.142
MI	4 (0.3)	0 (0.0)	0 (0.0)	2 (0.5)	2 (0.5)	0.260	0.073
IDR	128 (8.7)	9 (2.5)^a,b^	23 (6.3)^c^	45 (12.3)^a^	51 (13.9)^b,c^	<0.001	<0.001
Stroke	15 (1.0)	4 (1.1)	2 (0.5)	4 (1.1)	5 (1.4)	0.734	0.562

Values are *n* (%). *p* values are from chi-square test or Fisher exact test. *p* value for trend from Cochran-Armitage test. For the ^a^, ^b^, ^c^, and ^d^ symbols within the same row, identical symbols denote statistically significant differences between the corresponding groups after pairwise post hoc comparisons with Bonferroni correction. For patients who were lost to follow-up, their outcome data were imputed using the last observation carried forward method. μCTI, combined 3V-μQFR and CTI index; MACCE, major adverse cardiovascular and cerebrovascular events; Q, quartile; MI, myocardial infarction; IDR, ischemia-driven revascularization.

### Independent associations of μCTI and CTI with MACCE risk

To assess the independent associations of μCTI and CTI with MACCE risk, multivariable Cox PH regression models were established (Table 4). When analyzing μCTI as a continuous variable, in the model unadjusted for any confounders (Model 0), each standard deviation (SD) increase in μCTI was associated with a corresponding 72% increase in MACCE risk (HR = 1.72, 95% CI: 1.51–1.96). After sequentially adjusting for confounders, this association remained stable and even slightly strengthened, yielding an HR of 1.79 (95% CI: 1.54–2.07, *p* < 0.001) in the fully adjusted model. On the other hand, when μCTI was analyzed by quartile groups (with Q1 as the reference), across all four models, the MACCE risk was consistently and significantly higher in Q3 and Q4 compared to Q1. In the fully adjusted model (Model 3), the risk in Q3 was 3.46 times that in Q1 (HR = 3.46, 95% CI: 2.06–5.80), and the risk in Q4 was 4.02 times that in Q1 (HR = 4.02 95% CI: 2.36–6.87). Although the risk in Q2 showed an increasing trend (HR = 1.66), it did not reach statistical significance (95% CI: 0.95–2.92). Furthermore, a trend test across the quartile groups indicated that MACCE risk increased significantly with higher μCTI quartiles (P for trend < 0.001). Consistent with μCTI, in the fully adjusted model, CTI remained an independent predictor of MACCE regardless of the analytical approach (continuous or quartile). It is noteworthy that the risk associated with CTI did not increase monotonically with quartiles; the HR for Q3 was higher than that for the highest quartile (Q4). To ensure the stability of the multivariable Cox regression models, multicollinearity was assessed for all covariates included in the fully adjusted model. VIF analysis results (Supplementary Table S4) showed that all variables had VIF values below 5, indicating no severe multicollinearity issues.

**Table 4:** Multivariable Cox regression analysis of μCTI and CTI for predicting MACCE risk.

Indicator	Variable	Model 0	Model 1	Model 2	Model 3
		HR (95% CI)			
μCTI	Each-SD increase^a^	1.72 (1.51–1.96)*	1.70 (1.49–1.95)*	1.76 (1.53–2.02)*	1.79 (1.54–2.07)*
	μCTI Categories^b^				
	Q1	1 (Reference)			
	Q2	1.64 (0.94–2.86)**	1.63 (0.93–2.86)**	1.64 (0.93–2.87)**	1.66 (0.95–2.92)**
	Q3	3.38 (2.05–5.59)*	3.25 (1.96–5.41)*	3.33 (2.00–5.53)*	3.46 (2.06–5.80)*
	Q4	3.81 (2.32–6.26)*	3.73 (2.24–6.21)*	3.95 (2.36–6.62)*	4.02 (2.36–6.87)*
	*P-*trend	<0.001	<0.001	<0.001	<0.001
CTI	Each-SD increase^a^	1.61 (1.34–1.95)*	1.69 (1.37–2.09)*	1.69 (1.37–2.08)*	1.67 (1.34–2.10)*
	CTI Categories^b^				
	Q1	1 (Reference)			
	Q2	1.46 (0.86–2.49)**	1.44 (0.84–2.47)**	1.45 (0.85–2.47)**	1.47 (0.86–2.53)**
	Q3	3.21 (2.01–5.15)*	3.10 (1.92–5.01)*	3.10 (1.93–5.00)*	3.15 (1.94–5.13)*
	Q4	2.79 (1.72–4.51)*	2.91 (1.75–4.82)*	2.91 (1.76–4.81)*	2.92 (1.73–4.92)*
	*P-*trend	<0.001	<0.001	<0.001	<0.001

*p < 0.001; **p > 0.05.

a Each-SD increase: HR for each SD increment in μCTI.

b Categories: The first quartile (Q1) of μCTI/CTI was used as the reference group in all regression models.

Model 0: Unadjusted model.

Model 1: Adjusted for age and gender.

Model 2: Adjusted for Model 1 + hypertension, diabetes, smoking, previous MI, previous PCI and clinical diagnosis.

Model 3: Adjusted for Model 2 + LVEF, BMI, Scr, HbA1c, LDL-C, HDL-C and TC.

μCTI, combined 3 V-μQFR and CTI index; CTI, C-reactive protein-triglyceride glucose index; 3 V-μQFR, three-vessel μQFR; MACCE, major adverse cardiovascular and cerebrovascular events; HR, hazard ratio; CI, confidence interval; SD, standard deviation; LVEF, left ventricular ejection fraction; BMI, body mass index; Scr, serum creatinine; HbA1c, glycated hemoglobin; LDL-C, low-density lipoprotein cholesterol; HDL-C, high-density lipoprotein cholesterol; TC, total cholesterol.

Then we specifically analyzed a composite of harder endpoints (all-cause death, MI, and stroke), the association with μCTI was attenuated and no longer statistically significant (HR = 1.29, 95% CI: 0.98–1.71, *p* = 0.074) (Supplementary Table S3).

### Restricted cubic spline (RCS) analysis

**Figure 4:**
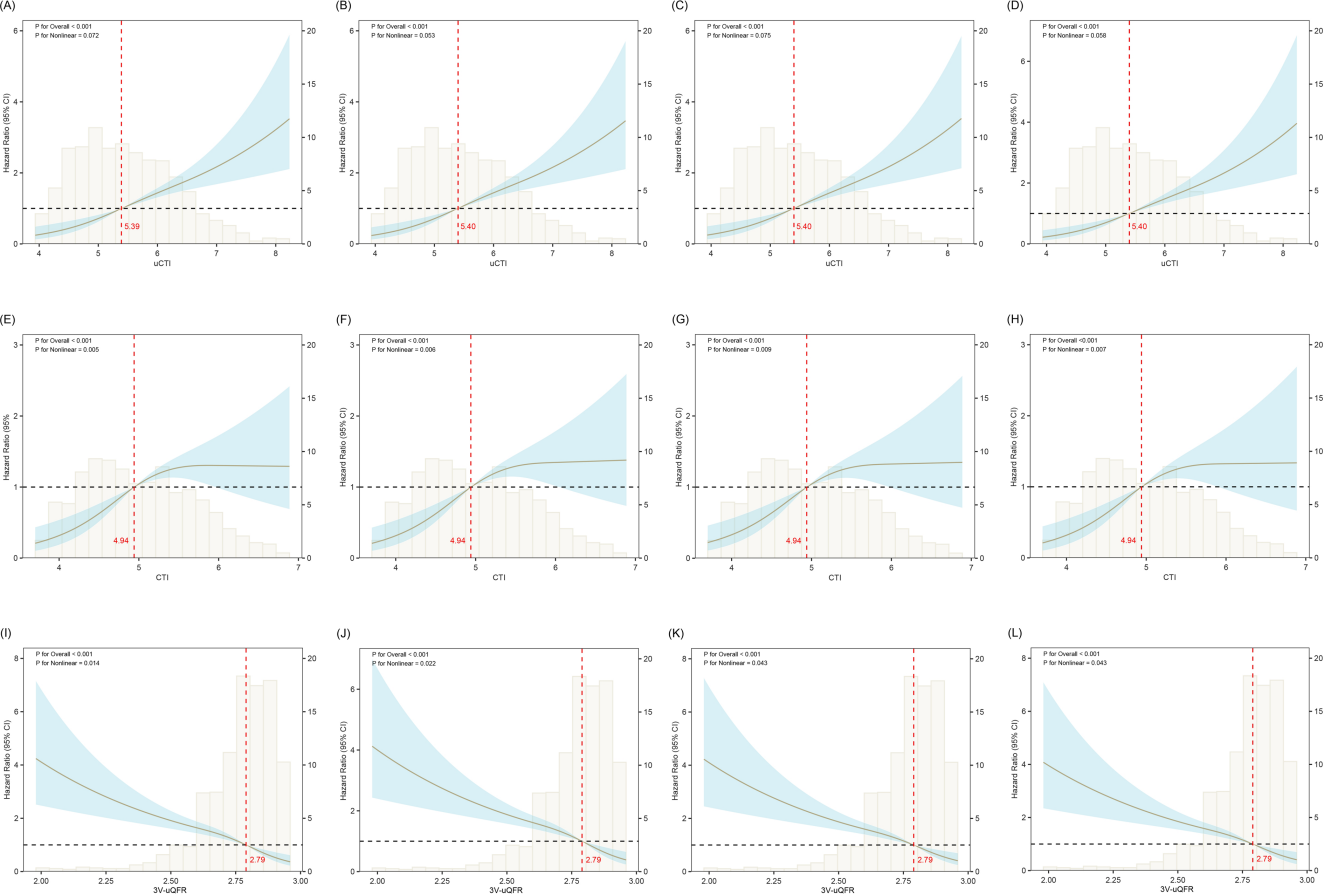
RCS analysis of the association between μCTI, CTI, 3 V-μQFR and MACCE risk. RCS curves show the dose–response relationships of μCTI, CTI, and 3 V-μQFR with MACCE risk. Model 0 **(A, E, I)**: Unadjusted model. Model 1 **(B, F, J)**: Adjusted for age and gender. Model 2 **(C, G, K)**: Adjusted for Model 1 + hypertension, diabetes, smoking, previous MI, previous PCI and clinical diagnosis. Model 3 **(D, H, L)**: Adjusted for Model 2 + LVEF, BMI, Scr, HbA1c, LDL-C, HDL-C and TC. RCS, restricted cubic spline; μCTI, combined 3 V-μQFR and CTI index; CTI, C-reactive protein-triglyceride glucose index; 3 V-μQFR, three-vessel μQFR; MACCE, major adverse cardiovascular and cerebrovascular events; MI, myocardial infarction; PCI, percutaneous coronary intervention; LVEF, left ventricular ejection fraction; BMI, body mass index; Scr, serum creatinine; HbA1c, glycated hemoglobin; LDL-C, low-density lipoprotein cholesterol; HDL-C, high-density lipoprotein cholesterol; TC, total cholesterol.

To explore the dose–response relationships between μCTI, CTI, 3 V-μQFR and MACCE risk, we performed RCS analysis with 3 degrees of freedom to visualize the associations (Figure 4). The results showed that in the univariable model, μCTI exhibited a linear positive correlation with MACCE (P for nonlinearity = 0.072), while CTI and 3 V-μQFR demonstrated J-shaped nonlinear associations (all P for nonlinearity < 0.05). These findings remained consistent in the partially and fully adjusted multivariable models.

### Subgroup analysis and interaction tests

**Figure 5:**
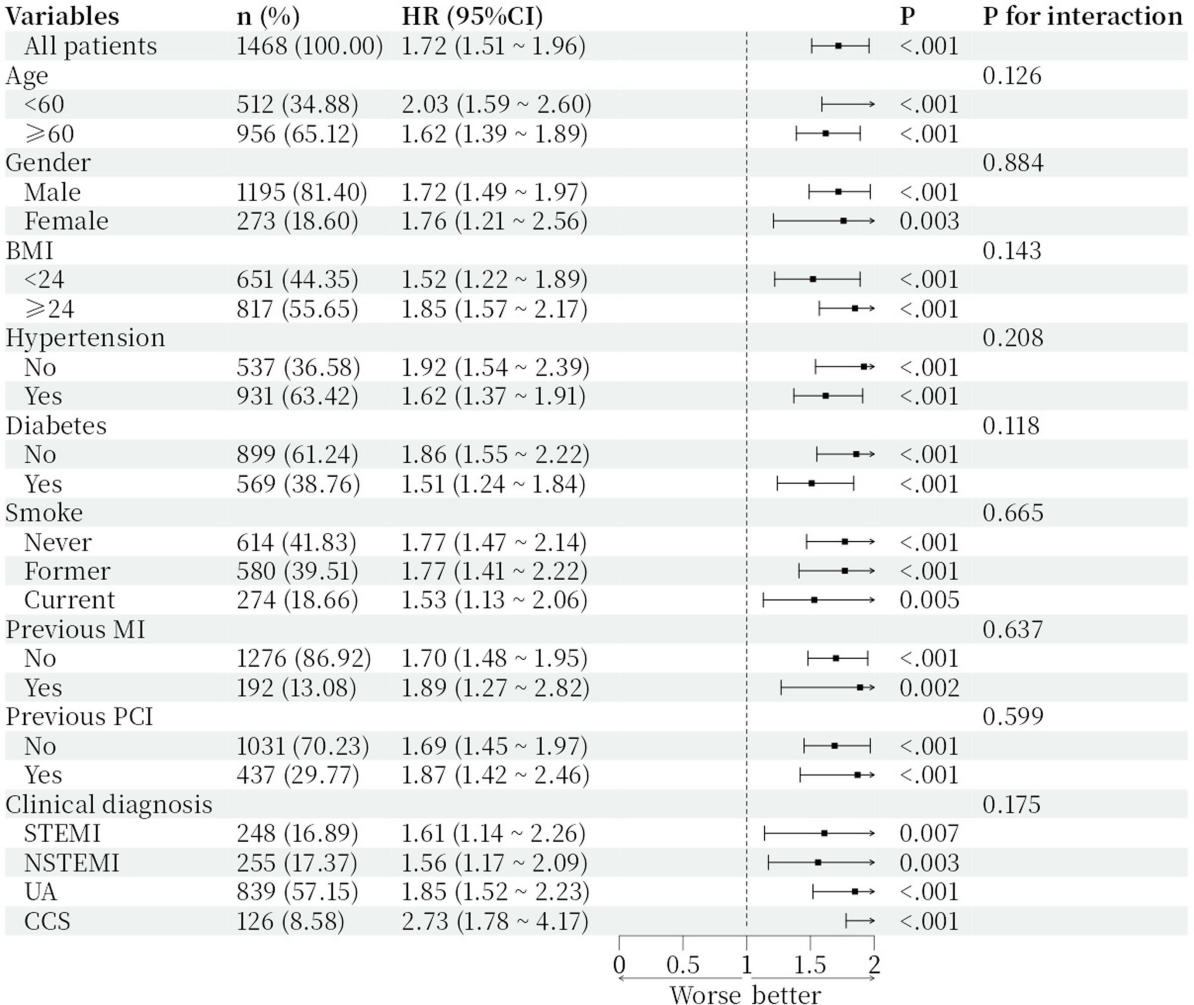
Subgroup analysis of the association between μCTI (per *SD* increase) and MACCE risk. Forest plot showing the *HR* and 95% *CI* for the association between each *SD* increase in μCTI and MACCE risk across predefined clinical subgroups. *P* for interaction > 0.05 for all subgroups, indicating no significant heterogeneity. *HR*, Hazard ratio; *CI*, Confidence interval; BMI, Body mass index; MI, Myocardial infarction; PCI, Percutaneous coronary intervention; STEMI, ST-segment elevation myocardial infarction; NSTEMI, Non-ST-segment elevation myocardial infarction; UA, Unstable angina; CCS, Chronic coronary syndromes; μCTI, Combined 3 V-μQFR and CTI index; 3 V-μQFR, Three-vessel μQFR; CTI, C-reactive protein-triglyceride glucose index; *SD*, Standard deviation; MACCE, Major adverse cardiovascular and cerebrovascular events.

To further examine the generalizability of the association between μCTI and MACCE risk, analyses were conducted across subgroups defined by different clinical characteristics (Figure 5). When μCTI was included as a continuous variable, each SD increase was significantly associated with an increased risk of MACCE across all predefined subgroups, encompassing age (<60 vs. ≥60 years), gender, BMI (<24 vs. ≥24 kg/m^2^), hypertension, diabetes, previous MI, previous PCI, and clinical diagnosis (all *p* < 0.05). However, the *p*-values for interaction tests across all subgroups were greater than 0.05, indicating that the strength of the association between μCTI and MACCE was consistent among populations with different ages, sexes, baseline comorbidities, and clinical types of CAD.

### Sample size calculation

Prior to conducting this study, we performed an a priori sample size calculation to ensure adequacy. The a priori sample size was calculated using G*Power version 3.1. A chi-square test (contingency table) was employed to estimate the required sample size for comparing MACCE incidence differences among μCTI quartile groups. The parameters were set as follows: significance level (*α*) = 0.05, power (1–*β*) = 0.90, degrees of freedom = 3, and a conservatively estimated effect size w = 0.20. The calculation yielded a minimum required sample size of 355. Accounting for a potential 10% loss to follow-up, at least 395 patients needed to be enrolled. This study ultimately successfully included 1,468 patients undergoing PCI, far exceeding the estimated minimum.

### Sensitivity analysis

To verify the robustness of the main analysis results, a series of sensitivity analyses were conducted. First, we assessed the core assumptions of the Cox PH models. Schoenfeld residual tests showed that μCTI itself, whether treated as a continuous or categorical variable, did not violate the PH assumption (Supplementary Figure S1, *p* = 0.965 and 0.217, respectively). Furthermore, the global test incorporating all covariates yielded *p*-values of 0.113 and 0.033 (Supplementary Table S5), indicating that the PH assumption was violated for the model based on μCTI quartiles. As detailed in Supplementary Table S5, this violation in the global test was primarily driven by three covariates: gender, previous MI, and previous PCI (each with *p* < 0.05). After removing these variables in Model 2 (Supplementary Table S6), the model’s predictive performance showed only a minimal change compared to fully adjusted Model 1, and the global test for this final model satisfied the PH assumption (Supplementary Table S7). Second, multigroup propensity score matching using nearest-neighbor matching was performed to balance baseline characteristics across the different μCTI groups (Supplementary Figure S2). Multivariable Cox regression analysis in this matched cohort (n = 1,377) showed that the associations of μCTI Q3 and Q4 groups with higher MACCE risk remained significant, consistent with the conclusions of the primary analysis (Supplementary Table S8). Third, the main analysis was repeated in cohorts that excluded specific patient subgroups. After excluding patients with severely reduced left ventricular ejection fraction (LVEF <40%), the significant association between μCTI and MACCE risk persisted (Supplementary Table S9). Moreover, after excluding patients with baseline chronic total occlusion or acute total occlusion lesions, this association became even more pronounced (e.g., in the fully adjusted model, HR for Q4 = 5.55, 95% CI: 2.80–11.00) (Supplementary Table S10). When the analysis was restricted to patients who achieved functional complete revascularization, although the HRs for Q3 and Q4 exhibited a non-monotonic pattern in the quartile-based analysis, the trend test across quartiles remained statistically significant (P for trend < 0.001) (Supplementary Table S11). Finally, a worst-case scenario imputation was applied to outcome data for patients lost to follow-up, assuming that all such patients experienced a MACCE event. Under this most conservative analytical scenario, the results of the primary analysis remained consistent with our main findings (Supplementary Table S12).

## Discussion

We proposed and validated for the first time a combined index, μCTI, which integrates systemic metabolic-inflammatory load (CTI) with global coronary physiological burden (3 V-μQFR). The principal finding of this study was that μCTI was an independent predictor of MACCE in post-PCI patients and provided additional risk stratification. Furthermore, extensive sensitivity and subgroup analyses confirmed the robustness of the primary findings.

A recent study based on the CHARLS database (27) used K-means clustering analysis and found that participants with persistently high and increasing CTI levels had a significantly increased risk of cardiovascular disease compared to those with persistently low CTI levels. Furthermore, cumulative CTI demonstrated better predictive performance than the TyG index or CRP alone. A study based on NHANES (18) found that CTI significantly predicted cardiovascular mortality (HR = 2.28, 95% CI: 1.69–3.24) and all-cause mortality (HR = 2.14, 95% CI: 1.76–2.55), and was associated with total cardiovascular disease incidence. These findings further support the advantage of metabolic-inflammatory composite index in risk stratification.

In addition to systemic metabolic and inflammatory markers, the assessment of local coronary functional status is equally crucial for comprehensively understanding the risk and prognosis of patients with CAD. Studies have shown that the second-generation Murray’s law-based μQFR exhibits diagnostic efficacy comparable to conventional dual-view QFR in identifying hemodynamically significant stenoses (32). Substantial clinical evidence indicates that PCI guided by physiological indices such as QFR can significantly improve patients’ long-term prognosis (33–36). Multiple clinical studies have confirmed that, using fractional flow reserve (FFR) as a reference, QFR demonstrates high sensitivity and specificity in diagnosing functional myocardial ischemia (20–22). The FAVOR III China trial series (37–39) further demonstrated that a QFR-guided revascularization strategy significantly reduced the risk of the primary composite endpoint compared to traditional angiography guidance. Consequently, several major international guidelines and consensus statements now recommend QFR as a reliable alternative tool for functional assessment (40–42).

Our study proposed the novel composite index μCTI, which integrates two distinct pathological assessments: the coronary physiological burden quantified by 3 V-μQFR primarily reflects existing physiological abnormalities that can induce myocardial ischemia and serves as a direct basis for guiding revascularization; whereas the systemic metabolic-inflammatory load assessed by CTI dynamically reflects the systemic pathophysiological activity progression and instability. Among patients with similar coronary physiological burden, a higher μCTI level may reflect more active systemic metabolic-inflammatory status, which could be associated with greater plaque vulnerability and a higher long-term event risk. This approach may provide complementary prognostic information beyond single-dimensional assessment using either CTI or 3 V-μQFR alone. Rather than implying a direct mechanistic pathway, μCTI should be interpreted as a composite risk marker reflecting different dimensions of residual risk after PCI. Further prospective studies are needed to determine whether μCTI-guided risk assessment can improve clinical decision-making or patient outcomes.

It is noteworthy that although μCTI showed no significant difference in discriminative ability compared to 3 V-μQFR, μCTI still exhibited significant improvement in reclassification capability, indicating its incremental value in further stratifying patient risk levels. Additionally, the observation that 3 V-μQFR showed higher predictive performance over CTI may suggest that, for patients with established CAD, the local functional ischemic burden in the heart exerts a more direct driving effect on mid-term events than the systemic metabolic-inflammatory state. Although μCTI showed improved predictive performance compared with CTI, the TyG index, and hs-CRP, its overall AUC remained limited-to-moderate. Therefore, the statistical improvement observed in discrimination and risk reclassification should not be interpreted as definitive evidence of substantial clinical benefit in routine practice.

Our study confirms a significant nonlinear association between CTI and MACCE risk. Moreover, when CTI was integrated with coronary physiological load indices, the resulting μCTI demonstrated a smoother linear relationship with outcome events. After adjustment for confounding variables, each standard deviation increase in μCTI was associated with a 79% rise in MACCE incidence. The association between μCTI and MACCE should be interpreted in the outcome structure. As shown in our analysis (Supplementary Table S3), the association of μCTI with a composite hard endpoint was attenuated and no longer statistically significant. This suggests that the prognostic utility of μCTI may be most salient for predicting ischemia-driven revascularization, which constitutes a major and clinically relevant component of MACCE in contemporary PCI cohorts. The IDR-dominant structure of MACCE may be explained by two main factors. First, IDR was the most frequent component of MACCE in this cohort, whereas death, myocardial infarction, and stroke occurred less frequently, limiting the statistical power to detect differences in these hard endpoints. Second, μCTI incorporates residual coronary physiological burden assessed by 3 V-μQFR, which is closely related to residual ischemia and the likelihood of repeat revascularization. Therefore, μCTI may be more sensitive in identifying patients prone to recurrent ischemia or clinically driven revascularization than those at risk of irreversible hard events. Accordingly, the prognostic value of μCTI in the present study should be interpreted primarily in relation to recurrent ischemia and repeat revascularization, while its association with hard clinical endpoints requires further validation in larger cohorts with longer follow-up.

Importantly, multiple analytical results in this study converge to support a concise and feasible risk stratification threshold for μCTI, facilitating its clinical translation. The Hommel-adjusted multiple comparison test of Kaplan–Meier curves, Bonferroni-corrected pairwise comparisons, and multivariable Cox regression analyses consistently indicated that MACCE risk did not differ significantly within the lower (Q1 vs. Q2) or the higher (Q3 vs. Q4) μCTI quartiles. Nonetheless, the higher-tier groups (Q3 and Q4) exhibited a significant and graded increase in risk relative to the lower-tier groups (Q1 and Q2), supporting a dichotomy in risk stratification. In addition, the median μCTI value of 5.40 closely aligns with the optimal cutoff (5.50) derived from ROC analysis, validating μCTI = 5.40 as a potential binary threshold for distinguishing between lower-risk and moderate-to-high-risk patients. In practical clinical application, this threshold may identify patients at significantly elevated risk of MACCE, thereby providing a clear, actionable quantitative basis for implementing more intensive monitoring and secondary prevention strategies.

The evaluation of 3 V-μQFR is based on existing coronary angiographic images, analyzed through dedicated post-processing software without requiring any additional consumables or medications during the procedure, and can also be performed offline after the intervention (21). Consequently, the ability of μCTI to provide incremental prognostic information at very low additional cost makes it highly attractive from a health economics perspective in secondary prevention.

### Study limitations

Our study has several limitations. First, this was a single-center observational study. Although prospective data collection and multivariable adjustment were employed, residual confounding cannot be entirely ruled out, and causality cannot be directly inferred. Therefore, multicenter studies and external validation in independent cohorts are warranted to confirm the prognostic value and clinical applicability of μCTI. Second, the study population was limited to patients undergoing PCI; therefore, extrapolating the conclusions to other populations requires caution. Third, the relationship between μCTI and hard endpoints such as death and myocardial infarction requires further validation in larger studies with longer follow-up. Furthermore, recent individual studies have raised differing perspectives on the clinical utility of QFR (43, 44), potentially due to factors such as operator experience (45).

## Conclusion

This study proposed and validated for the first time a novel integrated index, μCTI. It combines CTI to reflect systemic metabolic-inflammatory status and 3 V-μQFR to assess global coronary physiological burden. The results demonstrate that μCTI serves as an independent predictor of MACCE in patients after PCI. Compared with other indicators, the μCTI model showed improved overall performance in discrimination and risk reclassification. This integrated metric, at minimal additional cost, provides prognostic information beyond single-dimensional assessments. It holds promise for enabling finer residual risk stratification following revascularization and offers new evidence for formulating individualized secondary prevention strategies.

## Data Availability

The original contributions presented in the study are included in the article/Supplementary material, further inquiries can be directed to the corresponding authors.

## Glossary

CAD: Coronary artery disease
IR: Insulin resistance
TyG: Triglyceride-glucose
CRP: C-reactive protein
CTI: C-reactive protein-triglyceride glucose index
QFR: Quantitative flow ratio
μQFR: Murray’s law-based QFR
PCI: Percutaneous coronary intervention
MACCE: Major adverse cardiovascular and cerebrovascular events
3 V-μQFR: Three-vessel quantitative flow ratio
CFAF: Coronary functional adjustment factor
μCTI: combined 3 V-μQFR and CTI index
MI: Myocardial infarction
IDR: Ischemia-driven revascularization
ROC: Receiver operating characteristic
AUC: Area under the curve
NRI: Net reclassification improvement
IDI: Integrated discrimination improvement
HR: Hazard ratios
CI: Confidence intervals
VIF: Variance inflation factor
RCS: Restricted cubic spline
PH: Proportional hazards
RASi: Renin-angiotensin system inhibitors
SGLT2i: Sodium-glucose cotransporter 2 inhibitors
SD: Standard deviation
