# Supplementary material for: Prognostic value of a novel indicator integrating C-reactive protein-triglyceride glucose index and coronary physiological burden in patients undergoing PCI: a prospective cohort study
Source: Front Nutr. 2026 Jul 31;13:1874235. doi: 10.3389/fnut.2026.1874235 (PMC13473336; doi:10.3389/fnut.2026.1874235)
Supplement: Supplementary file 1 [file Table_1.DOCX]

**Supplementary materials**

**Table S1** Vessel-level baseline characteristics.

**Table S2** Baseline characteristics of participants grouped by study completion and loss to follow-up.

**Table S3** Multivariable Cox regression analysis of μCTI and the risk of a composite hard endpoint.

**Table S4** VIF for variables in the multivariable Cox regression models.

**Table S5**  Schoenfeld residual test for the Cox proportional hazards assumption.

**Table S6** Multivariable Cox regression analysis of μCTI and MACCE risk.

**Table S7**  Schoenfeld residual test for the Cox proportional hazards assumption.

**Table S8** Multivariable Cox regression analysis of μCTI and MACCE risk after multigroup PSM.

**Table S9** Multivariable Cox regression of μCTI and MACCE risk in patients with LVEF ≥ 40%.

**Table S10** Multivariable Cox regression of μCTI and MACCE risk after excluding total occlusions.

**Table S11** Multivariable Cox regression analysis of μCTI and MACCE risk in patients who achieved FCR.

**Table S12** Multivariable Cox regression of μCTI and MACCE risk with worst-case imputation.

**Fig. S1**  Schoenfeld residual test for the Cox proportional hazards assumption.

**Fig. S2** Standardized mean differences before and after multigroup PSM.

**Table S1** Vessel-level baseline characteristics

| **Coronary territories** | **Total (*N* = 4404)** | **LAD (*N* = 1468)** |  | **LCX (*N* = 1468)** | **RCA (*N* = 1468)** |
| --- | --- | --- | --- | --- | --- |
| MLD (mm) | 1.63 ± 0.68 | 1.42 ± 0.54 |  | 1.66 ± 0.68 | 1.83 ± 0.74 |
| RVD (mm) | 2.83 ± 1.14 | 2.69 ± 0.61 |  | 2.73 ± 1.27 | 3.09 ± 1.35 |
| Length of lesions (mm) | 21.61 ± 15.01 | 24.46 ± 14.70 |  | 18.57 ± 12.05 | 21.91 ± 17.33 |
| DS (%) | 42.14 ± 21.22 | 47.55 ± 22.99 |  | 38.60 ± 17.36 | 40.40 ± 21.95 |
| AS (%) | 62.85 ± 20.62 | 69.30 ± 17.77 |  | 59.26 ± 20.05 | 60.13 ± 22.28 |
| Pre-PCI QFR | 0.72 ± 0.31 | 0.61 ± 0.31 |  | 0.79 ± 0.27 | 0.75 ± 0.31 |

Data are presented as mean ± SD. LAD, left anterior descending; LCX, left circumflex; RCA, right coronary artery; MLD, minimal lumen diameter; RVD, reference vessel diameter; DS, diameter stenosis; AS, area stenosis; PCI, percutaneous coronary intervention; QFR, quantitative flow ratio.

**Table S2** Baseline characteristics of participants grouped by study completion and loss to follow-up

| **Characteristic** | **Overall (*N* = 1468)** | **Completed (*N* = 1420)** | | **Lose (*N* = 48)** | ***P* value** |
| --- | --- | --- | --- | --- | --- |
| **Clinical features** |  |  |  | |  |
| Age, years | 63.57 ± 10.79 | 63.54 ± 10.75 | 64.33 ± 11.99 | | 0.618 |
| Female, n(%) | 273 (18.60) | 265 (18.66) | 8 (16.67) | | 0.727 |
| BMI, kg/m2 | 24.49 ± 3.16 | 24.48 ± 3.18 | 24.85 ± 2.52 | | 0.431 |
| Diabetes, n(%) | 569 (38.76) | 548 (38.59) | 21 (43.75) | | 0.471 |
| Hypertension, n(%) | 931 (63.42) | 898 (63.24) | 33 (68.75) | | 0.436 |
| Previous MI, n(%) | 192 (13.08) | 188 (13.24) | 4 (8.33) | | 0.321 |
| Previous PCI, n(%) | 437 (29.77) | 425 (29.93) | 12 (25.00) | | 0.463 |
| Smoking, n(%) |  |  |  | | 0.502 |
| Never | 614 (41.83) | 590 (41.55) | 24 (50.00) | |  |
| Former | 274 (18.66) | 266 (18.73) | 8 (16.67) | |  |
| Current | 580 (39.51) | 564 (39.72) | 16 (33.33) | |  |
| Clinical diagnosis |  |  |  | | 0.004 |
| CCS, n(%) | 126 (8.58) | 121 (8.52) | 5 (10.42) | |  |
| UA, n(%) | 839 (57.15) | 820 (57.75) | 19 (39.58) | |  |
| NSTEMI, n(%) | 255 (17.37) | 248 (17.46) | 7 (14.58) | |  |
| STEMI, n(%) | 248 (16.89) | 231 (16.27) | 17 (35.42) | |  |
| **Laboratory/[examination](https://wwwn.cdc.gov/nchs/nhanes/search/datapage.aspx?Component=Examination&CycleBeginYear=2005) data** |  |  |  | |  |
| LDL-C (mmol/L) | 2.85 ± 1.24 | 2.83 ± 1.15 | 3.28 ± 2.86 | | 0.286 |
| TG (mmol/L) | 1.71 ± 1.24 | 1.70 ± 1.24 | 1.77 ± 0.97 | | 0.699 |
| TC (mmol/L) | 4.37 ± 1.29 | 4.37 ± 1.29 | 4.43 ± 1.29 | | 0.766 |
| HDL (mmol/L) | 1.07 ± 0.31 | 1.07 ± 0.31 | 1.09 ± 0.30 | | 0.707 |
| Scr (umol/L) | 77.85 ± 17.77 | 77.84 ± 17.79 | 78.29 ± 17.43 | | 0.862 |
| FBG | 6.18 ± 2.16 | 6.16 ± 2.15 | 6.59 ± 2.38 | | 0.180 |
| HbAlc (%) | 6.71 ± 1.44 | 6.71 ± 1.44 | 6.70 ± 1.46 | | 0.932 |
| LVEF (%) | 60.69 ± 11.07 | 60.78+11.06 | 58.02+11.12 | | 0.090 |
| hsCRP | 1.43 (0.52, 5.50) | 1.39 (0.52, 5.38) | 2.78 (0.51, 7.11) | | 0.112 |
| CTI | 5.02 ± 0.72 | 5.01 ± 0.72 | 5.21 ± 0.78 | | 0.063 |
| μCTI | 5.49 ± 0.91 | 5.48 ± 0.91 | 5.66 ± 0.81 | | 0.190 |
| **Medication** |  |  |  | |  |
| Statins, n(%) | 1453 (98.98) | 1406 (99.01) | 47 (97.92) | | 0.394 |
| Beta-blocker, n(%) | 1155 (78.68) | 1113 (78.38) | 42 (87.50) | | 0.129 |
| RASi, n(%) | 940 (64.03) | 911 (64.15) | 29 (60.42) | | 0.596 |
| CCB, n(%) | 487 (33.17) | 472 (33.24) | 15 (31.25) | | 0.773 |
| SGLT2i | 246 (16.76) | 239 (16.83) | 7 (14.58) | | 0.682 |
| **Interventional data** |  |  |  | |  |
| Angiographic disease severity |  |  |  | | 0.955 |
| 1-vessel disease | 1083 (73.77) | 1048 (73.80) | 35 (72.92) | |  |
| 2-vessel disease | 315 (21.46) | 304 (21.41) | 11 (22.92) | |  |
| 3-vessel disease | 70 (4.77) | 68 (4.79) | 2 (4.17) | |  |
| 3V-μQFR | 2.76 ± 0.17 | 2.76 ± 0.17 | 2.76 ± 0.12 | | 0.803 |

Data are presented as mean ± SD, median (IQR), or n (%). Abbreviation as in Table 1.

**Table S3** Multivariable Cox regression analysis of μCTI and the risk of a composite hard endpoint

| Variable | Number(%) | Unadjusted model | | Adjusted model | |
| --- | --- | --- | --- | --- | --- |
|  |  | HR (95% CI) | P value | HR (95% CI) | P value |
| Each-SD increase^a^ |  | 1.42 (1.10-1.83) | 0.007 | 1.29 (0.98-1.71) | 0.074 |
| μCTI Categories^b^ |  |  |  |  |  |
| Q1 | 367 (25.0) | 1.00 (Reference) |  | 1.00 (Reference) |  |
| Q2 | 367 (25.0) | 0.83 (0.35-2.01) | 0.686 | 0.73 (0.29-1.81) | 0.492 |
| Q3 | 367 (25.0) | 1.74 (0.82-3.69) | 0.146 | 1.88 (0.86-4.10) | 0.115 |
| Q4 | 367 (25.0) | 1.86 (0.89-3.92) | 0.100 | 1.53 (0.66-3.55) | 0.319 |

Each-SD increase^a^: HR for each SD increment in μCTI.

μCTI Categories^b^: The first quartile (Q1) of μCTI was used as the reference group in all regression models.

Adjusted model: Adjusted for age, gender, hypertension, diabetes, smoking, previous MI, previous PCI, clinical diagnosis, LVEF, BMI, Scr, A1c, LDL,HDL and TC.

Abbreviation as in Table 4.

**Table S4** VIF for variables in the multivariable Cox regression models

| Characteristic | Df | μCTI | | quartiles of μCTI | |
| --- | --- | --- | --- | --- | --- |
|  |  | GVIF | GVIF^(1/(2*Df)) | GVIF | GVIF^(1/(2*Df)) |
| μCTI/quartiles of μCTI | 1 | 1.272 | 1.128 | 1.289 | 1.043 |
| Age | 1 | 1.206 | 1.098 | 1.206 | 1.098 |
| Gender | 1 | 1.661 | 1.289 | 1.662 | 1.289 |
| Clinical diagnosis | 3 | 1.416 | 1.060 | 1.418 | 1.060 |
| HBP | 1 | 1.095 | 1.046 | 1.096 | 1.047 |
| Diabetes | 1 | 1.675 | 1.294 | 1.679 | 1.296 |
| Smoke | 2 | 1.567 | 1.119 | 1.574 | 1.120 |
| Previous MI | 1 | 1.343 | 1.159 | 1.343 | 1.159 |
| Previous PCI | 1 | 1.373 | 1.172 | 1.375 | 1.172 |
| BMI | 1 | 1.129 | 1.063 | 1.132 | 1.064 |
| LDL | 1 | 4.166 | 2.041 | 4.175 | 2.043 |
| TC | 1 | 4.408 | 2.100 | 4.424 | 2.103 |
| HDL | 1 | 1.232 | 1.110 | 1.233 | 1.111 |
| Scr | 1 | 1.257 | 1.121 | 1.256 | 1.121 |
| HbAlc | 1 | 1.715 | 1.309 | 1.701 | 1.304 |
| LVEF | 1 | 1.239 | 1.113 | 1.239 | 1.113 |

Assessment of multicollinearity among covariates included in the fully adjusted Cox regression models (Model 3) for continuous μCTI and μCTI quartiles. A GVIF value < 5 indicates no severe multicollinearity.VIF, variance inflation factor; Df, degrees of freedom; GVIF, generalized variance inflation factor; Other abbreviations are shown in Table 1.

**Table S5**  Schoenfeld residual test for the Cox proportional hazards assumption

| Characteristic | Df | μCTI | | μCTI quartiles | |
| --- | --- | --- | --- | --- | --- |
|  |  | chisq | P Value | chisq | P Value |
| μCTI/μCTI quartiles | 1/3 | 0.00189 | 0.965 | 4.44533 | 0.217 |
| Global | 19/21 | 27.20496 | 0.100 | 34.33445 | 0.033 |
| Age | 1 | 0.04807 | 0.827 | 0.03180 | 0.859 |
| Gender | 1 | 6.25339 | 0.012 | 5.94384 | 0.015 |
| Clinical diagnosis | 3 | 1.75415 | 0.625 | 1.59319 | 0.661 |
| HBP | 1 | 0.88964 | 0.346 | 0.87239 | 0.350 |
| Diabetes | 1 | 0.95366 | 0.329 | 1.04819 | 0.306 |
| Smoke | 2 | 2.68345 | 0.261 | 2.90046 | 0.235 |
| Previous MI | 1 | 6.66805 | 0.010 | 6.95474 | 0.008 |
| Previous PCI | 1 | 6.49862 | 0.011 | 6.65073 | 0.010 |
| BMI | 1 | 0.00213 | 0.963 | 0.01169 | 0.914 |
| LDL | 1 | 0.19069 | 0.662 | 0.16186 | 0.687 |
| TC | 1 | 0.12944 | 0.719 | 0.08019 | 0.777 |
| HDL | 1 | 2.39990 | 0.121 | 2.98669 | 0.084 |
| Scr | 1 | 0.01321 | 0.909 | 0.02547 | 0.873 |
| HbAlc | 1 | 0.00756 | 0.931 | 0.00314 | 0.955 |
| LVEF | 1 | 0.01063 | 0.918 | 0.00259 | 0.959 |

Global and covariate-specific Schoenfeld residual test results. P > 0.05 suggests the proportional hazards assumption is met. df, degrees of freedom; chisq, chi-square statistic. Other abbreviations are shown in Table 1.

**Table S6** Multivariable Cox regression analysis of μCTI and MACCE risk

| Variable | Model 1 | | Model 2 | |
| --- | --- | --- | --- | --- |
|  | HR (95% CI) | P value | HR (95% CI) | P value |
| Each-SD increase^a^ | 1.79 (1.54-2.07) | <.001 | 1.78 (1.54-2.06) | <.001 |
| μCTI Categories^b^ |  |  |  |  |
| Q1 | 1.00 (Reference) |  | 1.00 (Reference) |  |
| Q2 | 1.66 (0.95-2.92) | 0.076 | 1.65 (0.94-2.89) | 0.082 |
| Q3 | 3.46 (2.06-5.80) | <.001 | 3.43 (2.04-5.75) | <.001 |
| Q4 | 4.02 (2.36-6.87) | <.001 | 4.01 (2.35-6.83) | <.001 |

Each-SD increase^a^: HR for each SD increment in μCTI.

μCTI Categories^b^: The first quartile (Q1) of μCTI was used as the reference group in all regression models.

Model 1: Completely adjusted.

Model 2: Adjusted for age, hypertension, diabetes, smoking, hyperlipidemia, clinical diagnosis, LVEF, BMI, Scr, A1c, LDL, HDL and TC.

Other abbreviations are shown in Table S3.

**Table S7**  Schoenfeld residual test for the Cox proportional hazards assumption

| Characteristic | Df | μCTI | | μCTI quartiles | |
| --- | --- | --- | --- | --- | --- |
|  |  | chisq | P Value | chisq | P Value |
| μCTI/μCTI quartiles | 1/3 | 0.01099 | 0.92 | 4.449168 | 0.22 |
| Global | 16/18 | 10.27586 | 0.85 | 17.651361 | 0.48 |
| Age | 1 | 0.04692 | 0.83 | 0.032417 | 0.86 |
| Clinical diagnosis | 3 | 1.74940 | 0.63 | 1.586150 | 0.66 |
| HBP | 1 | 0.86709 | 0.35 | 0.851226 | 0.36 |
| Diabetes | 1 | 0.94545 | 0.33 | 1.030097 | 0.31 |
| Smoke | 2 | 2.81353 | 0.24 | 2.965231 | 0.23 |
| BMI | 1 | 0.00105 | 0.97 | 0.009034 | 0.92 |
| LDL | 1 | 0.16160 | 0.69 | 0.145142 | 0.70 |
| TC | 1 | 0.12201 | 0.73 | 0.079894 | 0.78 |
| HDL | 1 | 2.46216 | 0.12 | 3.041536 | 0.08 |
| Scr | 1 | 0.01349 | 0.91 | 0.028302 | 0.87 |
| HbAlc | 1 | 0.00272 | 0.96 | 0.000949 | 0.98 |
| LVEF | 1 | 0.01353 | 0.91 | 0.004528 | 0.95 |

Abbreviations are shown in Table S6.

**Table S8** Multivariable Cox regression analysis of μCTI and MACCE risk after multigroup PSM

| Variable | Number(%) | Unadjusted model | | Adjusted model | |
| --- | --- | --- | --- | --- | --- |
|  |  | HR (95% CI) | P value | HR (95% CI) | P value |
| Each-SD increase^a^ |  | 1.85 (1.60-2.15) | <.001 | 1.88 (1.61-2.20) | <.001 |
| μCTI Categories^b^ |  |  |  |  |  |
| Q1 | 330 (24.0) | 1.00 (Reference) |  | 1.00 (Reference) |  |
| Q2 | 360 (26.1) | 1.62 (0.90-2.89) | 0.106 | 1.63 (0.91-2.93) | 0.010 |
| Q3 | 360 (26.1) | 3.40 (2.01-5.74) | <.001 | 3.40 (1.99-5.81) | <.001 |
| Q4 | 327 (23.7) | 3.92 (2.32-6.61) | <.001 | 4.00 (2.30-6.94) | <.001 |

Each-SD increase^a^: HR for each SD increment in μCTI.

μCTI Categories^b^: The first quartile (Q1) of μCTI was used as the reference group in all regression models.

Adjusted model: Adjusted for age, gender, hypertension, diabetes, smoking, previous MI, previous PCI, clinical diagnosis, LVEF, BMI, Scr, A1c, LDL,HDL and TC.

PSM, propensity score matching. Other abbreviations are shown in Table S3.

**Table S9** Multivariable Cox regression of μCTI and MACCE risk in patients with LVEF ≥ 40%

| Variable | Number(%) | Unadjusted model | | Adjusted model | |
| --- | --- | --- | --- | --- | --- |
|  |  | HR (95% CI) | P value | HR (95% CI) | P value |
| Each-SD increase^a^ |  | 1.73 (1.51-1.99) | <.001 | 1.83 (1.57-2.14) | <.001 |
| μCTI Categories^b^ |  |  |  |  |  |
| Q1 | 351 (25.3) | 1.00 (Reference) |  | 1.00 (Reference) |  |
| Q2 | 349 (25.2) | 1.62 (0.91-2.89) | 0.098 | 1.64 (0.92-2.92) | 0.097 |
| Q3 | 346 (25.0) | 3.26 (1.94-5.48) | <.001 | 3.32 (1.95-5.67) | <.001 |
| Q4 | 339 (24.5) | 3.82 (2.29-6.38) | <.001 | 4.16 (2.39-7.24) | <.001 |

Each-SD increase^a^: HR for each SD increment in μCTI.

μCTI Categories^b^: The first quartile (Q1) of μCTI was used as the reference group in all regression models.

Adjusted model: Adjusted for age, gender, hypertension, diabetes, smoking, previous MI, previous PCI, clinical diagnosis, LVEF, BMI, Scr, A1c, LDL, HDL and TC.

Abbreviation as in Table S3.

**Table S10** Multivariable Cox regression of μCTI and MACCE risk after excluding total occlusions

| Variable | Number(%) | Unadjusted model | | Adjusted model | |
| --- | --- | --- | --- | --- | --- |
|  |  | HR (95% CI) | P value | HR (95% CI) | P value |
| Each-SD increase^a^ |  | 1.77 (1.52 -2.06) | <.001 | 1.89 (1.57-2.28) | <.001 |
| μCTI Categories^b^ |  |  |  |  |  |
| Q1 | 302 (26.9) | 1.00 (Reference) |  | 1.00 (Reference) |  |
| Q2 | 282 (25.2) | 2.30 (1.16-4.58) | 0.018 | 2.30 (1.15-4.62) | 0.019 |
| Q3 | 273 (24.4) | 4.15 (2.19-7.89) | <.001 | 4.16 (2.15-8.06) | <.001 |
| Q4 | 264 (23.6) | 5.30 (2.83-9.95) | <.001 | 5.55 (2.80-11.00) | <.001 |

Each-SD increase^a^: HR for each SD increment in μCTI.

μCTI Categories^b^: The first quartile (Q1) of μCTI was used as the reference group in all regression models.

Adjusted model: Adjusted for age, gender, hypertension, diabetes, smoking, previous MI, previous PCI, clinical diagnosis, LVEF, BMI, Scr, A1c, LDL, HDL and TC.

Abbreviation as in Table S3.

**Table S11** Multivariable Cox regression analysis of μCTI and MACCE risk in patients who achieved FCR

| Variable | Number(%) | Unadjusted model | | Adjusted model | |
| --- | --- | --- | --- | --- | --- |
|  |  | HR (95% CI) | P value | HR (95% CI) | P value |
| Each-SD increase^a^ |  | 1.64 (1.33-2.02) | <.001 | 1.76 (1.37-2.25) | <.001 |
| μCTI Categories^b^ |  |  |  |  |  |
| Q1 | 344 (28.2) | 1.00 (Reference) |  | 1.00 (Reference) |  |
| Q2 | 320 (26.3) | 2.07 (1.11-3.88) | 0.023 | 2.10 (1.11-3.95) | 0.022 |
| Q3 | 297 (24.4) | 3.92 (2.19-7.01) | <.001 | 3.84 (2.10-7.01) | <.001 |
| Q4 | 257 (21.1) | 3.25 (1.77-5.96) | <.001 | 3.47 (1.80-6.70) | <.001 |

Each-SD increase^a^: HR for each SD increment in μCTI.

μCTI Categories^b^: The first quartile (Q1) of μCTI was used as the reference group in all regression models.

Adjusted model: Adjusted for age, gender, hypertension, diabetes, smoking, previous MI, previous PCI, clinical diagnosis, LVEF, BMI, Scr, A1c, LDL, HDL and TC.

FCR, functional complete revascularization. Other abbreviations are shown in Table S3.

**Table S12** Multivariable Cox regression of μCTI and MACCE risk with worst-case imputation

| Variable | Number(%) | Unadjusted model | | Adjusted model | |
| --- | --- | --- | --- | --- | --- |
|  |  | HR (95% CI) | P value | HR (95% CI) | P value |
| Each-SD increase^a^ |  | 1.63 (1.44-1.84) | <.001 | 1.69 (1.44-1.87) | <.001 |
| μCTI Categories^b^ |  |  |  |  |  |
| Q1 | 367 (25.0) | 1.00 (Reference) |  | 1.00 (Reference) |  |
| Q2 | 367 (25.0) | 1.45 (0.90-2.33) | 0.129 | 1.51 (0.93-2.45) | 0.093 |
| Q3 | 367 (25.0) | 2.82 (1.84-4.32) | <.001 | 2.90 (1.86-4.51) | <.001 |
| Q4 | 367 (25.0) | 3.27 (2.15-4.98) | <.001 | 3.34 (2.13-5.24) | <.001 |

Each-SD increase^a^: HR for each SD increment in μCTI.

μCTI Categories^b^: The first quartile (Q1) of μCTI was used as the reference group in all regression models.

Adjusted model: Adjusted for age, gender, hypertension, diabetes, smoking, previous MI, previous PCI, clinical diagnosis, LVEF, BMI, Scr, A1c, LDL, HDL and TC.

Abbreviation as in Table S3.

**Fig. S1**  Schoenfeld residual test for the Cox proportional hazards assumption. Schoenfeld residual plots for the continuous μCTI variable (A) and categorical μCTI quartiles (B). A non-horizontal pattern would indicate violation of the proportional hazards assumption.

**Fig. S2** Standardized mean differences before and after multigroup PSM. Bar plot displaying differences for all covariates before (red squares) and after (blue circles) multigroup PSM across μCTI quartiles. PSM, propensity score matching; μCTI, combined 3V-μQFR and CTI index.
